# Sensible Gentleman

**Published:** 1858-05

**Authors:** 


### SENSIBLE GENTLEMAN.

"I PROMISED a particular friend of mine to send him a bottle of the kind of dyspeptic medicine I have been taking; but I thought it would do him more good if I would send you the money, and have you to send him the JOURNAL OF HEALTH—as it would cost the same for the bottle as for one year's subscription; and I think, if he will read the JOURNAL, he will derive considerably more benefit, and throw physic to the dogs."

So says a subscriber to the Journal. We wish it could be known throughout this land, that no medicine ever swallowed for dyspepsia has had any other lasting effect than to aggravate the malady, when mainly relied on. Yet it is so much easier to swallow drugs than to make careful observations and self-denials, that even intelligent people allow themselves to fall helplessly into the arms of any transparent ignoramus who can muster the courage to write or speak a deliberate and unblushing falsehood. The only radical cure for dyspepsias and neuralgias is the proper adaptation of suitably nutritious food, and judicious exercise.

